# Tracing a new path in the field of AI and robotics: mimicking human intelligence through chemistry. Part II: systems chemistry

**DOI:** 10.3389/frobt.2023.1266011

**Published:** 2023-10-17

**Authors:** Pier Luigi Gentili, Pasquale Stano

**Affiliations:** ^1^ Department of Chemistry, Biology, and Biotechnology, Università degli Studi di Perugia, Perugia, Italy; ^2^ Department of Biological and Environmental Sciences and Technologies (DISTeBA), University of Salento, Lecce, Italy

**Keywords:** chemical artificial intelligence, chemical robots, artificial neural networks, proteins, DNA, fuzzy logic, Bayesian inference

## Abstract

Inspired by some traits of human intelligence, it is proposed that *wetware* approaches based on molecular, supramolecular, and systems chemistry can provide valuable models and tools for novel forms of robotics and AI, being constituted by soft matter and fluid states as the human nervous system and, more generally, life, is. Bottom-up mimicries of intelligence range from the molecular world to the multicellular level, i.e., from the Ångström ( $10^{-10}$ meters) to the micrometer scales ( $10^{-6}$ meters), and allows the development of unconventional chemical robotics. Whereas conventional robotics lets humans explore and colonise otherwise inaccessible environments, such as the deep oceanic abysses and other solar system planets, chemical robots will permit us to inspect and control the microscopic molecular and cellular worlds. This article suggests that systems made of properly chosen molecular compounds can implement all those modules that are the fundamental ingredients of every living being: sensory, processing, actuating, and metabolic networks. Autonomous chemical robotics will be within reach when such modules are compartmentalised and assembled. The design of a strongly intertwined web of chemical robots, with or without the involvement of living matter, will give rise to collective forms of intelligence that will probably reproduce, on a minimal scale, some sophisticated performances of the human intellect and will implement forms of “general AI.” These remarkable achievements will require a productive interdisciplinary collaboration among chemists, biotechnologists, computer scientists, engineers, physicists, neuroscientists, cognitive scientists, and philosophers to be achieved. The principal purpose of this paper is to spark this revolutionary collaborative scientific endeavour.

## 1 Introduction

The research field of AI and Robotics has the declared objective of building intelligent machines and the implicit purpose of understanding what intelligence is, especially that shown by humans (Russell and Norvig, 2010), and according to certain paradigms. Although,as humans, we have been meditating on our intelligence, at least, since the birth of philosophy in ancient Greece (i.e., since the far VI century BC), we are still missing a universally accepted definition. Minsky (2006) coined the expression “suitcase word” for terms like intelligence, which is multifaceted. Human intelligence has many features, and its definition might depend on the context. Attempting to define it from the Complexity Science viewpoint, it might be proclaimed that intelligence is an amazing emergent property of the human nervous system (HNS; Gentili, 2021a). As such, it is expected that by assembling artificial complex systems that are architectural mimicry of the HNS, some performances of human intelligence should pop up. This idea is supported by the methodology that the cognitive scientists Gallistel and King (2009) and the neuroscientist Marr (2010) have proposed to understand any biological complex system. Their methodology requires analysing the HNS at three distinct levels. The first analysis is at the “computational level” and requires determining inputs, outputs, and the system’s computations. The second one is at the “algorithmic level” and involves formulating algorithms reproducing the previously found computations. The final analysis is performed at the “implementation level” and requires designing and implementing artificial mechanisms that run the formulated algorithms. This methodology has been embraced by chemists who try to mimic some performances of human intelligence using chemistry: they contribute to AI and Robotics by tracing a new path and developing unconventional Chemical Artificial Intelligence (CAI; Gentili, 2013). In the first part of this series (Gentili and Stano, 2023b), it has been demonstrated—according to the current experimental investigations—that molecules and supramolecules can be exploited to reproduce some elementary functions, such as “sensing,” “computing,” “communicating,” and “working.” In this second part, it will be shown that it is feasible to imitate more elaborate performances of human intelligence by recurring to Systems Chemistry. Systems Chemistry refers to the design of complex mixtures of properly chosen chemical compounds that can give rise to emergent properties, i.e., properties that go beyond the sum of the characteristics of the system’s individual constituents (Ashkenasy et al., 2017). The emergent properties of artificial chemical mixtures, designed according to the three-level analysis mentioned above, can show some primary forms of intelligence.

## 2 Chemical robotics

**FIGURE 1:**
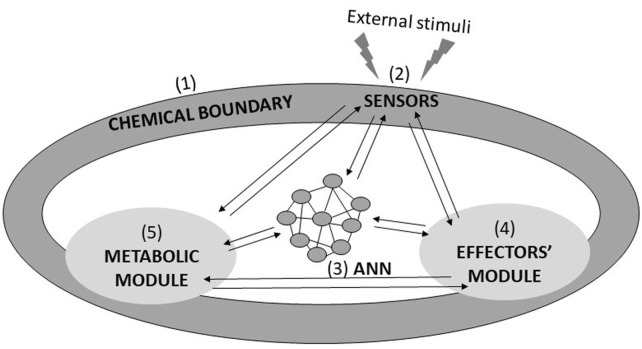
Sketch showing the required fundamental elements of a Chemical Robot, whose prototype is any unicellular organism. A Chemical Robot is a chemical system confined by (1) a chemical boundary along which are deployed (2) the sensors; it includes (3) the Artificial Neural Network (ANN), (4) the Effectors’ module, and (5) the metabolic unit. All the listed components must be strongly intertwined, conferring to the Chemical Robot the power of perceiving, generating knowledge, planning and acting: in a nutshell, autonomy and intelligence. It should perform analytical processing, practical, and regulatory functions (Zhang et al., 2019).

The ultimate and ambitious goal of Systems Chemistry in the field of CAI is the development of the so-called Chemical Robotics (also named Molecular Robotics or Cybernetics; Hagiya et al., 2014; Murata et al., 2022). Chemical Robots are supposed to be autonomous and adaptive molecular assemblies, confined through a membrane, and provided with four other modules, which are also the prerogatives of every living cell (see Figure 1). The first is the sensory module, made of the molecular and supramolecular logic gates described in part I (Gentili and Stano, 2023b). The sensory module guarantees data collection related to the surrounding environment’s features and the robot’s internal state. The sensory data must be processed by an artificial neural network module, which also takes the decisions. The resolutions trigger the action of the effectors’ module, which is constituted by proper assemblies of molecular machines. Finally, the intelligent activities of any Chemical Robot should be sustained by a metabolic unit. An obvious application of Chemical Robots can be identified in nanomedicine, i.e., as smart drug-delivery (or drug-producing) agents. In such a scenario, miniaturized Chemical Robots will be implanted in living beings where they will interplay with living cells and organelles to perform bio-medical actions, such as releasing a drug in the right place and at the right moment. They should become “Medical Doctors within individual cells” (Shapiro, 2012) or auxiliary elements of the natural immune systems. These autonomous elements should be programmable molecular computing devices capable of computing synthetic biopolymers and releasing them in response to environmental inputs. It is convenient, then, to describe their design and operation in the computationalist paradigm, here based on and related to the central dogma of molecular biology (Crick, 1970). All the potential performances of any living being are encoded in the chemical composition of a polymer, which is the DNA. Its information is transcribed into a specific sequence of the polymer RNA through RNA polymerase. Finally, it is translated into a peculiar poly-aminoacidic sequence (i.e., a protein) through the ribosome. This naturally occurring way of processing information has striking similarities with the working principle of the universal computing machine proposed by Alan Turing in 1936. The Turing machine (Turing, 1936) works on an infinitely long tape (which can be a potentially unbounded DNA or RNA polymer). It contains a head (it can be RNA polymerase or the ribosome) that can write and read symbols while moving forward and backwards on the tape. A Chemical Robot, driven by a DNA- or RNA-based Turing machine, can face any solvable computing problem and regulate biomolecular processes *in vivo* because it can interact directly with the biochemical environment, offering new avenues for gene therapy (Varghese et al., 2015). Chemical Robots may also be employed to safeguard the environment and face problems related to energy and food supplies (Murata et al., 2013). Chemical Robot’s performances depend on the compartmentalisation of its modules and how well they are assembled and integrated. The perfect epitome is represented by any unicellular microorganism. Hence, the development of Chemical Robotics can be rightly embedded within the broad field of synthetic biology. In particular, the Chemical Robot we are referring to can be fabricated through the bottom-up approach (Luisi, 2002; Guindani et al., 2022), i.e., by assembling all the molecular elements required to ensure the expected functions. As a result, the so-called *synthetic (or artificial) cells* are obtained.

### 2.1 Artificial neural networks

A pivotal role in a Chemical Robot is played by its artificial neural network module. As mentioned above, it can be a DNA- or RNA-based Turing machine, or it might be more similar to the human brain. The human brain is a complex network of about $80\times 10^{9}$ nerve cells (Herculano-Houzel, 2009), whose outstanding performances derive from its massive parallelism. The parallelism is generated by the remarkable connectivity of the neurons, each having up to 10^4^ synapses. Each neuron is a non-linear switching dynamical system with an intrinsic operating frequency that can reach the kHz range. It is reasonable to think of approaching some elementary computational functions of the human brain by devising Artificial Neural Networks (ANN). ANNs are biologically inspired networks wherein the nodes, i.e., the neural analogues, send signals between each other through weighted edges, representing synaptic links. The high interconnectivity of any ANN guarantees massively parallel computation and high defect tolerance (Ha and Ramanathan, 2011). ANNs allow the implementation of adaptive computation. Adaptive computational systems are capable of pattern recognition, content addressable memory, control systems, medical diagnosis, and all those problems that are now prerogatives of machine learning algorithms (Alpaydin, 2020). ANNs are traditionally implemented in software. However, it is more energetically convenient to implement them in hardware. Neuromorphic engineering in hardware is developed mainly through memristive devices, which can change their conductance in response to electrical pulses. Memristors are made of different material classes ranging from magnetic alloys, metal oxides, and chalcogenides to 2D van der Waals or organic materials (Christensen et al., 2022). Molecular and Systems Chemists propose alternative implementations of neural surrogates and ANNs in wetware.

Proteins are valuable neural surrogates at the molecular level (Bray, 1995). They constitute the basic information-processing elements of the complex intracellular molecular reaction network that controls the physiology of living cells and organisms. In the signalling network of every cell, a protein, which links a substrate in its active site and transforms it chemically into a specific product, acts as a computational node. The information is encoded in the three-dimensional structure of the molecules, and it is primarily communicated through diffusion and, when possible, through advection of the liquid solution. The simple molecules that work as inputs and outputs of all the protein-based information-processing events establish most of the links among the proteins. Sometimes, the link is direct and implies protein-protein associations. In cellular signalling networks, a high degree of nonlinearity, mimicking that of neural networks, is guaranteed by allosteric proteins. A protein is allosteric when its activity towards a peculiar substrate is affected by other molecules (called effectors) that link to other sites of the same protein (Dokholyan, 2016). The effectors can accelerate or decelerate the reaction governed by the protein. Sometimes, the chemical species produced by a protein can play as its own effector and positive or negative feedback actions can be implemented. The protein-based signalling networks are the brains of living cells because they process the sensory data related to the external environment and internal cellular state and take the appropriate course of action, triggering specific epigenetic events, i.e., activating and/or inhibiting the expression of specific genes in DNA (Roederer, 2005).

The other two fundamental macromolecular ingredients of every cell, i.e., DNA and RNA, have also been proposed as basic elements for implementing ANNs. DNA and RNA exist as strands made of sequences of four smaller molecules known as nucleotides: Adenine (A), thymine (T), guanine (G), and cytosine (C) in the case of DNA, and in RNA, uracil (U) substitutes T. Each nucleotide is complementary to another specific nucleotide due to the number and strength of hydrogen bonds that are established: A is complementary to T (or U in the case of RNA), and C to G. Based on this peculiar chemical complementarity, it is possible to exploit partially doubled-stranded molecules of DNA or RNA as neural surrogates. When a single-stranded DNA (or RNA) molecule finds a partially double-stranded molecule with a complementary sequence, they bind, causing the partially doubled-stranded molecule to shed the strand that was previously on it. The single-stranded DNA molecule that successfully binds to the partially double-stranded DNA molecule acts as the system’s input, whereas the strand kicked off by the bonding DNA molecule acts as the system’s output. Once released, an output strand can be input by interplaying with another partially double-stranded DNA molecule. ANNs built using DNA or RNA hybridisation reactions, in which two complementary single-stranded DNA or RNA molecules bond to form a double-stranded molecule, can run challenging computations (Qiu et al., 2013). They can be employed to solve NP-complete problems (Cox et al., 1999), such as the Travelling Salesman Problem (Adleman, 1994), or they can be appropriate for the recognition of variable patterns (Cherry and Qian, 2018).

Neural surrogates can be alternatively implemented through proper reactive chemical systems that, when maintained far from the thermodynamic equilibrium, reproduce the dynamics of real neurons (Przyczyna et al., 2020). They replicate the oscillatory, chaotic, or excitable dynamic regimes of real neurons (Izhikevich, 2007). A well-known instance is the Belousov-Zhabotinsky (BZ) reaction, which can proceed in oscillatory, chaotic, or excitable regimes depending on the physicochemical conditions (Epstein and Pojman, 1998). Information is encoded in the concentrations of specific chemical species, which determine the electric potential values and the UV-visible absorption properties of the macroscopic solutions. Distinct neural surrogates of the type of the BZ reaction can communicate through diffusion or advection, as in the case of the proteins, or, alternatively, through the propagation of chemical waves. Communication becomes ultrafast when the light transmitted or emitted by the neural surrogates is used as signals. Optical signals are quickly transferred among physically distant neural surrogates (Gentili et al., 2017; Gentili et al., 2018). When all the neural surrogates that are reciprocally linked are in their oscillatory regime, it is possible to implement Spiking Neural Networks (SNNs). SNNs, also called Oscillatory Neural Networks (ONNs), constitute a promising computing paradigm. It is analog because the information is encoded on the frequency of the oscillators and the phase relations between oscillators. It is low-power because the information is not encoded in the amplitude of the signals, and therefore the signal amplitude can be very low, reducing power consumption (Corentin et al., 2021). ONNs are helpful for recognising variable patterns. Memorised patterns are synchronised oscillatory states in which neurons fire periodically with certain relations between their phases (Hoppensteadt and Izhikevich, 2000). ONNs also promise to contribute to cutting-edge computing that should go beyond Moore’s law by devising alternative architectures to current electronic computers. Moore’s law plays a relevant role when we face the computation of hard problems, i.e., when we face exponential problems (or NP problems) with large dimensions. We can expect to solve accurately NP-problems only if we devise ultrafast computing machines. Since Moore’s law will stop holding soon because transistors are made of a few atoms, the only way to solve NP problems having large dimensions is to design novel computing architectures, revolutionizing the Von Neumann architecture of current electronic computers (Csaba and Porod, 2020). Such hard problems can also be faced through a top-down strategy (Gorecki, 2022): After choosing a proper computing chemical medium, how the input and the output information are encoded must be fixed. The properties of the medium must be controlled by some adjustable macroscopic parameters. Within this strategy, the values of parameters for which the output gives the most accurate solution must be found. To perform such optimization, several training examples are needed to verify the accuracy of computation performed by the medium.

## 3 Sophisticated reasonings: fuzzy logic and Bayesian inference

**FIGURE 2:**
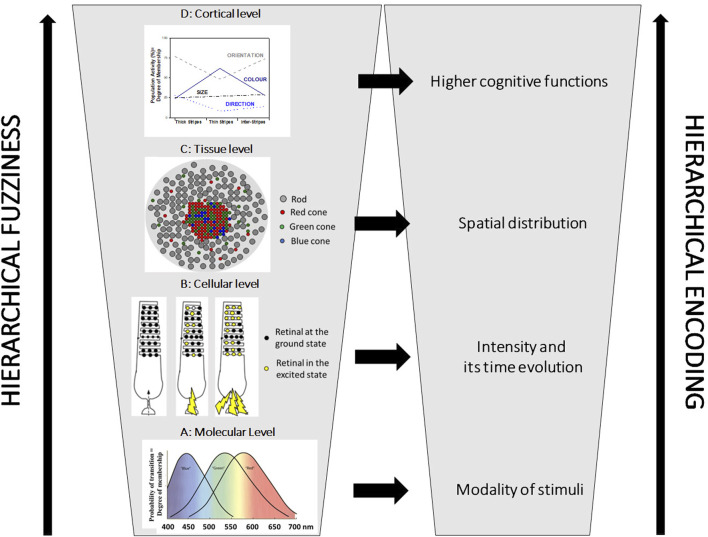
The hierarchical fuzziness and encoding in human vision. In the bottom left part, graph **(A)** shows the absorption bands of the three retinals that belong to the so-called “Blue,” “Green,” and “Red” cones. They are three molecular fuzzy sets granulating the visible spectral region. They allow encoding the modality of the light stimuli. Graph **(B)** shows the schematic structure of a rod with many retinals in its outer segment: lights (represented by the yellow lightning) with the same spectral compositions but different intensities excite different amounts of the retinals. The intensity of the light is encoded as the degree of membership to the cellular fuzzy set (see the right panel). Graph **(C)** represents a sketch of the retina. The spatial distribution of the light stimuli is encoded at the tissue level through the array of cellular fuzzy sets on the retina (see the right panel). Plot **(D)** shows that all three compartments (thick stripes, thin stripes, and inter-stripes) of the cortical visual area V2 participate, at different degrees, in the experience of colour, orientation, size, and direction of the objects that humans see [data extrapolated from Gegenfurtner (2003)]. The three compartments are intrinsically fuzzy. They are connected to the fuzzy Cortical Columns of the visual area V1.

Chemical Robots will never be capable of tightly reproducing the power of human minds if they rely only upon Boolean logic. They might need to take rational decisions in environments dominated by uncertainty, partiality and relativity of truth. In these situations, other types of logic and inference are required. For instance, fuzzy logic is particularly helpful in the case of partially true statements and context-dependent decisions. It reproduces how humans make decisions using syllogistic statements of the type IF…, THEN… expressed through the natural language (Zadeh, 1997). Adjectives employed in the formulation of syllogistic statements are fuzzy sets granulating the numerical values of the variables. The meaning of any adjective is context-dependent, likewise the position and shape of fuzzy sets. A reason why fuzzy logic is a good model of human capability to compute with words has been attributed to some intrinsically fuzzy features of the HNS (Zadeh, 2002; Gentili, 2014). The information of any physicochemical stimulus is encoded hierarchically (Figure 2 shows the specific case of human vision): the modality at the molecular level, the intensity and its time-evolution at the cellular level, and the spatial distribution at the sensory organ level. These features of any stimulus are encoded as fuzzy information because the collections of molecules and cells involved in any sensory system work as ensembles of fuzzy sets, granulating the attributes of the physicochemical variables. The afferent neurons connecting the sensory cells to the brain operate further granulations of the variables through their peculiar receptive fields (Gentili, 2018). All the higher-level intelligent activities, such as sensory perception, knowledge generation, planning, and decision-making, are believed to take place in the neocortex, which comprises almost $30\times 10^{9}$ billion neurons and about 10^14^ synapses. Both anatomically and functionally, the cerebral cortex is describable as a hive of cortical columns (Mountcastle, 1997; Rakic, 2008). Since humans usually navigate uncertain conditions, the reasoning seems highly consistent with Bayesian probabilistic inference (Pouget et al., 2013). If the symbol CC represents the cortical columns activated in either a perception or a decision or an action H , within the context c , then, the posterior probability $p\left(H|CC,c\right)$ , according to the Bayes’ formula, is given by:

$$p\left(H|CC,c\right)=\frac{p\left(CC|H,c\right)p\left(H,c\right)}{p\left(CC,c\right)}$$

It is a combination of the current information, represented by the likelihood $p\left(CC|H,c\right)$ , and past information, embodied in the prior probability $p\left(H,c\right)$ , normalized by the plausibility $p\left(CC,c\right).$ All the terms appearing in Bayes’ formula can be interpreted as fuzzy information because experimental evidence demonstrates that the cortical columns work as fuzzy sets (Gegenfurtner, 2003; Gentili, 2021b): distinct cerebral events belong to the several cortical columns at different degrees. The brain as a whole seems to have a highly distributed functionality with many different areas contributing to every its function (Wells, 2005).

Such interpretation of sophisticated human reasoning blazes a trail for its chemical implementation. Chemistry will allow the imitation of the hierarchical fuzziness and information encoding of the HNS through a bottom-up approach. In the first part of this series of two papers (Gentili and Stano, 2023b), it has already been mentioned that single fuzzy sets can be implemented at the molecular level through those compounds that exist as ensembles of conformers (conformers are molecules that differ just in the 3D arrangement of their constitutive atoms). Any conformational collection has context-dependent features: the conformers’ identity and relative abundance (Gentili and Perez-Mercader, 2022). Mixing properly chosen molecular fuzzy sets enlarge the power of information encoding.

Suppose the intermingled molecular fuzzy sets are sensitive to the same physicochemical variables. In that case, they can carry out the granulation and graduation of the variables, like the three retinals (the “Blue,” “Green,” and “Red” ones) shown in Figure 2A. The mimicry of human colour vision at the level of the retina, implemented through mixtures of photochromic compounds, which absorb different portions of the UV and originate specific absorption bands in the visible region, has allowed extending human vision to the ultraviolet (Gentili et al., 2016).

When the molecular fuzzy sets, which are mixed in the same solution, participate in different chemical reactions and give rise to a chemical web because they share reagents and products, they allow the implementation of chemical fuzzy neural networks. Suppose these webs are recurrent because they include feedback actions. In that case, they become the chemical counterparts of the neuro-fuzzy algorithms that are good at modelling non-linear causal relationships, predicting aperiodic time series, and recognising variable patterns (Gentili and Stano, 2022; Braccini et al., 2023). The feedback actions that can alter the strength of the reciprocal connections confer learning abilities to the chemical network.

Higher cognitive functions can be achieved by hierarchically increasing the chemical fuzzy neural networks’ complexity. In synthetic cells, distinct modules playing different functions can be assembled through their compartmentalization, likewise in living cells (Gentili and Stano, 2023a). One step further towards the hierarchical implementation of chemical artificial intelligence can be taken by designing networks of synthetic cells. Their performances will depend on their network’s architecture, how the artificial cellular nodes communicate, and the adaptability of the reciprocal links. When the edges are strong enough, a sort of swarm or collective intelligence (Couzin, 2007; Watson and Levin, 2023) exhibiting Bayesian inference might emerge. Such powerful webs of synthetic cells might also establish strong connections with living cells and originate the so-called Internet of Bio-Nano Things (IoBNTs; Stano et al., 2023). The hybrid and collective intelligence of the IoBNTs promises to have a plethora of applications (Akyildiz, et al., 2015; Kuscu and Unluturk, 2021), such as diagnosis and therapies for human health and control and cleaning in natural ecosystems or urban areas. In the IoBNTs, even two- (Kagan et al., 2022) or three-dimensional cultures of human brain cells (brain organoids; Smirnova et al., 2023) might be involved. Brain organoids can more easily recapitulate the histoarchitecture and functionality of the fuzzy cortical columns. Therefore, it is reasonable to envisage that such IoBNTs will approach the power of human intelligence more closely to process complex information based on uncertain and context-dependent data, with the extraordinary energetic efficiency of the human brain (Herculano-Houzel, 2012).

## 4 Conclusive remarks

The potentialities of CAI presented in this article, and the previous one (Gentili and Stano, 2023b) are summarized in Table 1. Moving from Molecular to Supramolecular and finally to Systems Chemistry, the complexity of the problems domain that can be faced apparently increases. Although Chemical AI and robotics are still in their infancy, undoubtedly, it is worth pursuing their development. Among the most impressive achievements of traditional AI and robotics are those pushing robots where humans cannot arrive: the exploration of the marine abysses and the colonization of other planets. Chemical AI and robots can help humans to explore another space poorly investigated so far, i.e., the molecular world. As the same Kurzweil (2014) proclaimed, the “colonization” of the molecular world “will provide tools to effectively combat poverty, clean up the environment, overcome diseases, and extend human longevity.” Massively distributed intelligent chemical robots will greatly expand our memories and sensory and computational abilities. The authors hope these perspectives on Chemical AI and robotics (parts I and II) will spark a productive interdisciplinary collaboration among chemists, biotechnologists, physicists, computer scientists, engineers, neuroscientists, cognitive scientists, philosophers, and biologists. The development of inanimate intelligent chemical systems through a bottom-up approach will have not only remarkable technological repercussions on our societies but will probably unveil that outstanding event that occurred about 4.5 billion years ago, and that was the appearance of life on Earth: a sort of phase transition (Solé et al., 1996; Perez-Mercader, 2004) from an inanimate world devoid of agents to the appearance of the first chemical systems capable of exploiting matter and energy to “handle” information and pursue goals.

**TABLE 1:** Lists of the problems that can be faced by exploiting solutions that emerge from CAI.

CAI Solutions	Problems domain
Molecular logic gates	Probes of the microscopic world
Molecular machines	Effector Modules of Chemical Robots
Chemical robots	Auxiliary elements of the immune system: protect and cure humans. Medical Doctor in a cell: diagnosis and therapy
	Suppliers of energy, food, and protection of the environment
Artificial neural networks	Adaptive computation: pattern recognition, content addressable memory, control systems, medical diagnosis
Oscillatory neural networks	Recognition of variable patterns; beyond Moore’s law computing: facing NP-problems
DNA-based ANNs	NP-problems and recognition of variable patterns
Fuzzy molecules and macromolecules	Chemical words for a molecular language expressing partial truths and context-dependent decisions
Chemical fuzzy neural networks	Modelling and predicting non-linear causal events and recognizing variable patterns
IoBNT	Bayesian inference; diagnosis and therapies for human health; control and cleaning in natural ecosystems or urban areas
	Energy-efficient computing

## Data Availability

The original contributions presented in the study are included in the article/Supplementary Material, further inquiries can be directed to the corresponding author.
